# Using Research Agreements to Build Respectful, Publication-Grade Scholarly Relationships in Liberal-Arts Settings

**DOI:** 10.3389/fpsyg.2019.00197

**Published:** 2019-02-11

**Authors:** Lauren E. Bloomfield, Nicole S. Carver, Damian G. Kelty-Stephen

**Affiliations:** Department of Psychology, Grinnell College, Grinnell, IA, United States

**Keywords:** research agreement, undergraduate research, liberal arts college, education, syllabus

## Introduction

Collaboration with undergraduate students at a small liberal-arts college has accelerated our research programs. Liberal-arts students bring interdisciplinary flair—or sometimes just a fresh perspective not yet pigeonholed by post-graduate specialization. Liberal-arts students come from diverse backgrounds and reach beyond their comfort zones to try an eclectic mix of scholarly work. However, given the liberal-arts emphasis on collaboration, community, and compromise, these students bring a different set of sensibilities about work, accountability, and authorship than might appear in research-university laboratories designed to function as a well-oiled research machine (van der Wende, 2011; Kilgo et al., 2015; Lewis, 2018). So, generating publication-grade research with students at a liberal-arts college is as much about reflecting on science as one of many distinct ways of knowing as about designing experiments, collecting data, and disseminating that knowledge through publication.

The most rewarding faculty-student collaborations for our lab have been experiences that begin and end with space for students and faculty to learn together about science as a culture. Implementing a research agreement has become a best practice for our faculty-student collaborations in that it sets up an ideal atmosphere for producing publishable research. It has helped us to foster important discussions about scientific culture that help educate students about how to approach scientific work responsibly, respectfully, productively, and with the most rewarding learning outcomes. Indeed, scientific culture has its own values, and the blessing of research with liberal-arts undergraduates is the opportunity to reflect on how those values align with or diverge from those of other cultures.

## Opening Dialogues to Initiate Students Into the Cultural Foundation for Publishable Research

Undergraduate research experiences can be both immensely positive but also immensely challenging. We are privileged to have motivated students eager to embark on new intellectual journeys, journeys with the potential to shape and transform their entire educational trajectory. Then again, undergraduate education is not just an intellectual exercise but coincides with major life changes and challenges that follow naturally from young adults living on their own, very often for the first time, and learning how to manage a complex set of obligations (McKinsey, 2016; Tieken, 2016). Publishable research is no small responsibility to add to these circumstances. Whether the experience becomes professionally formative for each student, faculty mentors have the real liability that their own anxieties, plans, and concerns for research could easily upset the already challenging balance of student obligations and student wellness. No matter the course credit or wages that we offer students, the higher stakes of working toward publishable research outcomes in a professional academic laboratory leave students more prone to feelings of isolation, anxiety, stress, uncertainty, bewilderment, and disengagement than students experiencing the same work through a safer lower-stakes course-based research experience with a classroom full of peers (Rand, 2015; Shapiro et al., 2015; Barrow et al., 2016; Kobulnicky and Dale, 2016; Frantz et al., 2017; Kamangar et al., 2017).

For research with undergraduates to warrant any public communication, internal communication is crucial for making research a constructive, positive occasion for undergraduate students' growth and education. The very same institutional structures allowing faculty members to recruit undergraduate students also produce power differentials that can stifle open, constructive dialogue. Faculty may be poor judges of when constructive challenge of research has turned from opportunity for growth and self-discovery into harmful stressor, whether from insensitive faculty demands or from unspoken student decisions to compromise other professional goals or wellness. Ideally, research can help students drive their own academic narrative, but this entire benefit is lost when undergraduates are not yet in the habit of reflecting on their goals and efforts. Faculty are in a position to communicate explicitly with students about how to approach this research field and to model mutual respect as a clear and necessary counterweight to the power differential. Faculty have the expertise and the authority to set the tone for the research relationship, and students do not normally have the expectation to begin that discussion. The hope to engage in publishable research could become an unhealthy burden when faculty fail to set that tone.

Sounding out cultural foundations is as good as an initiation for students into publishable research as it is an opportunity for seasoned researchers to keep a fresh look on old habits and values. Science is slow to change by design, but it is important to compare expectations and values in the research lab with social changes or pressures in the broader world outside the lab—where our undergraduates come from Popejoy and Fullerton (2016), Gauchat and Andrews (2018), Ioannidis (2018). In a time when science is considering how it promotes respect for all participating members (e.g., National Academies of Sciences Engineering and Medicine, 2018), faculty might constructively reflect on what it means to make every new visitor to scientific culture feel respected and welcome, especially if these visitors are to be coauthors. It is an occasion to unpack implicit faculty values and to examine them explicitly for undergraduate students to understand at the outset of the project.

## Background for Research Agreements

Our idea for the research agreement grew naturally out of the concept of the course syllabus as an agreement, a long tradition in the pedagogical literature about setting expectations for faculty-student working relationships (Parkes and Harris, 2002; Habanek, 2005). This tradition of contract-like syllabi provokes mixed responses that we hoped to navigate intentionally as we developed the agreement. Bleak extremes risk manifesting as “commodification” of scholarly labor in which instructors offer graded credits in exchange for hours worked and efforts expended. These authoritarian syllabi enumerate rules for student conduct and threaten penalties, in the form of grade deductions or recrimination by the institutional administration (Agger and Shelton, 2017). Legal scholars have cautioned that the “contract” description is not just fraught with legal liability but is actually at odds with court decisions (Kauffman, 2014; Rumore, 2016). Despite discouraging the “contract” description, these legal perspectives have nevertheless encouraged the design of collaborative documents in which faculty and students work to outline expectations and responsibilities. And in fact, collaborative syllabus design in which faculty and students can negotiate on the terms has become an important part of recent attempts to make academia more inclusive and to help invite student investment in the learning process (Hudd, 2003; Hess, 2008; Shaw, 2009; Stocker and Reddad, 2013; Fornaciari and Lund Dean, 2014; Kaplan and Renard, 2015).

In this spirit of collaborative syllabus-like agreements, we start research collaboration with an in-person meeting. The eventual goal of the meeting is for both student and faculty to sign a potentially revised copy of the existing agreement before research work started, but crucially, the face-to-face dialogue aims to empower students to ask for clarification or propose amendments pending mutual agreement before signing. It is fully possible that, in the process of explaining the values that the faculty takes as self-evident, the student's line of questioning could help the faculty to see old values in new light. Students may get the benefit of an explanation of scientific culture, and faculty may get the benefit of letting the student perspectives help them discover that, maybe, some values are outdated or no longer suitable. But more certainly, this discussion lays a foundation for high standards and communication—a foundation essential for generating publishable work.

## Agreement: Aims and Content

The research agreement that we have piloted scaffolds better communication between faculty and students to launch the research collaboration and to support publishable work to follow. The agreement sets a tone of mutual respect from the outset particularly by acknowledging the joint-authorship expectation, allowing students to provide input for editing the document. This research-agreement framework acknowledges student questions and uncertainties about research as valuable points of concern for discussion rather than any sign of unsuitability for research. Such a framework empowers undergraduates to ask important questions and to open the faculty members' eyes to yet unimagined but meaningful new directions—both in pursuing or developing professional research and in running a laboratory. Constructive discussion and research is only viable if students respect themselves enough to be able to seek clarification or explanation from their faculty mentor. Empowered students might seem to some like an obstacle to forward progress for junior faculty feeling “publish or perish” pressures. However, we know of no evidence of student rebellion. And on the contrary, under the faculty-student power differential, we urge greater vigilance in minimizing the much more probable risk that high-stakes publication-grade research could easily press mentors into abusing student effort or diminishing student self-worth (Straus and Sackett, 2014; Vianden, 2015; Kibbe et al., 2016; Kobulnicky and Dale, 2016; Colbert-White and Simpson, 2017). There is plenty of room in organic collaboration for faculty to lead by example and by reasoned instruction, and treating research students less like hired labor and more like collaborators could actually be beneficial for meeting goals and for more creative research (Gornall et al., 2018).

Reflecting on both the press of deadlines and rigor and the benefits of publishable research, this agreement emphasizes both rights and responsibilities of students producing research. Rights might also be responsibilities, or vice versa, reflecting our hope that this open, social process of science works best when collaborators are honest about needs and respectful of others' needs as well (Barajas and Frossard, 2018). These points include but are not limited to

1. How to reflect about daily work (e.g., with updates to faculty indicating plans at the beginning of the day and a summary of progress and challenges at the end of the day).
2. How students will get the most out of research work by exercising good self-care (e.g., regular sleep, eating, and exercise).
3. How to reflect on internal communication within the lab, particularly insofar as each colleague's professional development requires mutual respect, regular information sharing, and sometimes critical feedback with their rights to disagree.
4. How to reflect on coauthorship as a shared privilege to participate in public communication.

The current agreement (available in its entirety as a Supplemental Material) aims to discuss long-range issues of authorship up front and before any research begins in order to reduce all subsequent ambiguity about expectations and roles (Roberts, 2017). It discourages sparse definitions of coauthorship as about generating enough words toward the final draft of a manuscript. Instead, it encourages the broader notion of coauthorship as a contribution to the communal effort from the initial phases of hypothesis development, through experimental design, through manuscript preparation and revision, and all the way past manuscript composition or submission to the responsible defense and accountability to respond intelligently to interested readers. The agreement is definitely biased to individual faculty's own particular scientific acculturation: different scientific subcultures will inevitably disagree somehow. However, the process of welcoming students to the scientific process with full disclosure and open dialogue about scientific values has been a fulfilling experiment in guaranteeing that all students get the best out of their brush with science. Publication or not, the research agreement builds a context that supports long-term professional relationships.

## Conclusion

The promise of publishing research falls unevenly across long-term goals of faculty and of student. The inevitable disparity between faculty goals and student goals opens up a potentially vast pitfall in which research collaborations could slip needlessly into personal strife and professional failures (Moskal et al., 2013; Moore et al., 2018; Niehaus and Wegener, 2018). Student should have full view of the stakes and the larger setting of concerns in which research labs produce work. The agreement also gives the faculty the opportunity to express their goals and make sure they are either equal to the student's goals or in separate but parallel alignment (e.g., meeting departmental goals of providing research training). The co-authored research agreement offers a safe and mutually respectful context in which faculty and students can reflect on shared and unshared goals. Enhancing students' sense of professionalism, control, and ownership leads to a stronger commitment to seeing a project through from data collection to publication (Araujo et al., 2018; Cavanagh et al., 2018).

## Author Contributions

All authors listed have made a substantial, direct and intellectual contribution to the work, and approved it for publication.

### Conflict of Interest Statement

The authors declare that the research was conducted in the absence of any commercial or financial relationships that could be construed as a potential conflict of interest.
