# Supplementary material for: Using Research Agreements to Build Respectful, Publication-Grade Scholarly Relationships in Liberal-Arts Settings
Source: Front Psychol. 2019 Feb 11;10:197. doi: 10.3389/fpsyg.2019.00197 (PMC6378294; doi:10.3389/fpsyg.2019.00197)
Supplement: Supplementary file 1 [file Data_Sheet_1.PDF]

Research Agreement for Students in the D. Kelty-Stephen Research Lab

First of all, welcome. You are a valued student, and you would not be reading these words if I did not take you seriously as a scholar and as a colleague.

The reason for this agreement is that the research collaborator relationship can be extremely rewarding and extremely complex. Because similar complexity will color most non-classroom collaborations that you pursue here or beyond Grinnell, the experiment is worth an attempt. This agreement is not a legally binding document—it is an attempt to collaboratively determine our professional working relationship.

Engaging in research requires a wide range of your abilities and presses upon a wide range of your needs and constraints as a scholar. Classroom interactions with instructors require you to work on distinct and very narrow range of skills. However, research tends to draw on many more skills, from the technical details cultivated in a classroom environment to personal and professional skills that you have had to construct over a longer time scale and over a broader set of experiences than just one semester's class. In short, research requires much more of your personal investment because the demands of research are simply more multifaceted than a single academic skill. Ideally, this experience will give you a space to lead and to succeed, but the same space provides room for mistakes and failures and misunderstandings. The former aspect of research can be exciting and rewarding, but your greater personal investment in research can make the latter aspect of research challenging and stressful.

I would not have recruited you for research were I not completely happy for you to challenge me and to think for yourself and to convince me of what I might not understand. However, it is extremely important that you remember that I am responsible for your education and your wellness. In order to ensure that your efforts go towards a respectable product and to your academic/personal education, I will have to assert my authority, and you will have to accept specific responsibilities. The challenge for me here is to provide enough freedom for you to grow as an independent self-respecting scholar and avoid stifling you into the less empowered position as a subservient student. In the same spirit, however, I must also work to maintain that important sliver of inequality in our relationship wherein I am the supervisor who understands how research works and you are the student who is here to learn.

Both of us enter into this relationship with needs and expectations, and this agreement should ideally serve as an occasion to discuss these needs and expectations openly and peacefully. Later on, in the event of the inevitable misunderstandings and stumbles in research, this agreement may serve as a touchstone for any future discussions. You may consider it as much a resource for your own reference and guidance as a reminder of what I will expect in our future communications.

By reading and signing this document, you indicate your appreciation of the challenges following from this new sort of relationship, as well as your rights and responsibilities. Please read the below indications of rights and responsibilities, and please sign only if you accept the below terms or once we make satisfactory amendments. As a student whom I would hope to treat like a research colleague, you are responsible for helping us both/all to define the expectations and constraints for our collaboration.

Your Signature: ___________________________________ Date: ________________________________

My Signature: ____________________________________ Date: _________________________________

Page intentionally left blank (no need to initial)

As my collaborator, I expect you to act as co-owner of our work. That co-ownership entails certain rights and responsibilities. Some of these rights and responsibilities will overlap. Other rights are not responsibilities, and other responsibilities are not rights. Yet other rights can entail new responsibilities. I have attempted to inventory some of these items that warrant particular attention in what may be a new working relationship for you.

Primarily, you should know that everyone in the lab deserves respect no matter differences in identity. Research is difficult enough without anyone diminishing anyone else personally. We need to have the benefit of respectful acceptance of one another's differences, and it will help everyone to coexist peacefully and to collaborate constructively when we can communicate clearly about needs, about mistakes, and about how we can repair any damage done by any failures in perfectly professional mutual respect.

You have the right to be referred to respectfully according to you preferred gender pronouns

You have the right to work undisturbed by any discriminatory pressures, no matter whether those pressures are intentional or unintentional and no matter whether they apply explicitly to the work you have done or to you personally.

You have the responsibility to treat everyone else in the lab with respect and due consideration of their individual needs/preferences for that respect.

I really want you to understand that I need to know when you experience any failure of respect.

I need to know if you do not feel my respect for you—if I ever fail in this regard, I would like to know it so that I can make it up to you and stop doing anything you find disrespectful

I also need to know if you do not feel the full respect of any other students or collaborators involved in our work.

Your rights that ***are also*** responsibilities include but are not limited to:

to object to research ideas/plans/events on religious, moral, or ethical grounds

to free time, to pursue wellness, to sleep, to eat, and to exercise in the ways you prefer to be most healthy and effective.

to interrupt planned research projects when your wellness is not at the level you need for professional behavior, provided that you reschedule a later time in near future that will serve to make up the research time.

to admit confusion or misunderstanding, to ask for clarification, as repeatedly as necessary and with polite, professional interest.

I understand that there are many things all of us do not understand, and I understand that I can be quite terrible at explaining myself. However, if you do not honestly ask me to clarify, I cannot help you, and I can also not learn from you. My default is to think highly of you and to assume you do not need things clarified. If you withhold from me that you don't in fact understand, then I cannot help you like I would genuinely like to, and so withholding from me that you need help is only creating a potential for more hardship for both of us.

To separate feedback on your work from feedback on you.

My Initials _________Your Initials____________ Date________

I would like to state at the outset that I begin from the assumption that you are a wonderful person with many gifts to offer the world—otherwise, you would not be at Grinnell College. I will inevitably give positive feedback and also critical feedback. Important as it can be to feel good about positive feedback, it is an important life skill to maintain a detached posture with respect to critical feedback: you are not your work, and it is important for doing better work that you keep a boundary between criticism of your work and your own self-worth.

To get the most out of this learning experience by showing personal engagement and by reflecting openly about your experiences, especially about your expectations, how they are met or not met, and how you find your growth as a scientist resonating (or not) with your long-term goals/hopes/dreams.

For any of these rights that are also responsibilities, I reserve the right to call an in-person meeting to discuss any of these items that you may be neglecting. If you neglect any of these items, you do us both a disservice.

You are responsible for—which is another way of saying "I have the right to expect that you will do"—the following:

Reading e-mails and text messages in their entirety (including attachments) unless otherwise noted in the e-mail (e.g., that you may wait to read later) and, within the business week (Monday-Friday) during the academic year [or, for research over the summer when you should not have other distractions, within 48 hours within the business week], responding with answers to all questions, with your own new questions, and/or notice as to how long it will take you to generate answers and new questions.

Note that this responsibility does not apply when you are out of town, traveling, under doctor's care, or otherwise attending to your health and wellness.

Keeping track of instructions, whether that means taking notes in meetings or consulting e-mails or text messages.

When in doubt, following my instructions to the letter unless you find that circumstances require something new for the safety and care of yourself as well as of participants. *Change is unstoppable and undeniable. I trust you to do what is best in the moment and then to consult with me at your earliest convenience.*

Reporting to me ASAP with any changes to design or with any decisions to do anything different than what I instructed. I do not mind if plans change (even if it's immense or very quick), but it is essential that I know the change, the reason, and whether it warrants more permanent change/response.

Fielding my feedback on your work with the understanding that I hope to publish our work even if publishing is not a priority for you.

Remembering at all times that you are a student learning to do the very difficult thing called research. That is, remembering that you will often not see the light at the end of the tunnel or see the point for all steps in the process. Please trust my judgment if I recommend you to persist in what feels pointless, and please respect my longer view than yours.

Trusting me as the research adviser who is here to guide you to a research product that you can

be proud of and that you can be sure teaches you about the experience of doing scientific research. Your training so far makes you a good candidate for research training in my lab, and there are many points on which I will defer to your judgment, but you are not qualified to second guess my ability to point you on a constructive path.

Communicating promptly and politely any change in your willingness to participate in the research process or the preparation of scholarly products following from the research.

For any of these responsibilities, I reserve the right to call an in-person meeting to discuss any of these items that you may be neglecting.

My responsibilities to you are

To be respectful of your efforts, your time, and your status as a growing scholar as full of distinct skills as of areas for growth.

To respond promptly over e-mail or text within business hours, usually within 24 hours of your message. I may have a free moment and both hands with which to type a more thoughtful e-mail outside of business hours, but that would be the exception more than a rule.

To be available to meet in person and, if not available to meet within the next 24 hours, then at least to respond within 24 hours as to the next best times I can meet.

To give clear directions and to restate them as many times as you need and with the same level of respect each time.

To be accepting of your sensible interpretations of my instructions when you surprise me. Part of the fun and learning, for me as adviser, is from seeing how you can be creative while doing science.

To provide substantive feedback that will help you continue to make the most of your learning experience in scientific research.

For any of these responsibilities of mine to you, you have the right to call an in-person meeting to discuss any of these items that I may be neglecting.

By signing this agreement, you give me the right

To provide substantive feedback on your work, even if it is not always positive. You have the right to ask for clarification and for explanation of feedback, particularly with a view towards ensuring that you take my feedback as applying to your work and not to you. We shall work with the understanding that you are a great person, no matter what sort of work you do, and if you have trouble detaching criticisms of your work from criticisms of you, we will discuss the confusion in person.

To call a meeting to discuss any difficulties you have completing your responsibilities. You will know there is a concern because I will ask you to meet so we can revisit our agreement. I will give you this notice so you can brace yourself for a candid conversation about expectations and so that you can be ready with any documentation or notes that you would like to bring as well.

To review your communications with you and ask for your accounting of inconsistencies or gaps.

To give you a warning on the first and second occasion for these meetings.

To discontinue your project or your involvement in a group project on the third occasion for the

meeting, with the option for us to explore together whether you would benefit more from an alternate project less demanding and more supportive to your growth as a student.

To discontinue your participation in my lab if our attempts to resolve the aforementioned third occasion for revisiting our agreement does not prove successful OR if you respond to any bid for an agreement-revisiting meeting (first, second, or third) with hostility.

To alter your position in or to remove your name entirely from the author roster for any publication/poster/product in the event that you conduct yourself without proper respect for your student collaborators or to me.

For the most part, my research collaborations with students end positively and productively, and this work can be a stepping stone to further research with me or with a different lab.

That point brings me to concluding points I hope to make about how you and I might translate our collaborations into the long-term with publications and presentations. Here, we have arrived at the part about your potential participation as a young researcher who should have every interest in protecting your investment of efforts and in ensuring that you get the most out of this experience in the long term. There is more to gain from scientific research than monetary compensation, and in fact monetary compensation is the least of the gains.

Below is a list of your rights for how to represent your efforts and translate them into professional development beyond course credit and payment. Please bear in mind that, although these points are rights, they are rights that when taken advantage of trigger new responsibilities. These rights and consequent responsibilities appear below in pairs. Shortest two pairs appear first below, and the longest waits to the end to explain my expectations if you choose to pursue coauthorship.

The Right: to request recommendations and references from me for any subsequent employment. This point is actually most to your benefit, and it may be the most important contribution I can have to your career as a student.

The Responsibility: Taking advantage of this right entails that you provide me with a current resume as well as a paragraph describing the opportunity for which you would like my recommendation.

The Right: to propose new or different research, provided you also have a plan for carrying out the research with the stated/existing resources for funds.
The Responsibility: *Taking advantage of this right entails your responsibility as co-owner and co-author of resulting data and work.*

The Right: to coauthor posters/papers for conferences or journals on any of our work together
The Responsibility: *Taking advantage of this right entails that you share equally with all other coauthors in responsibility for the clarity and integrity of the work, that you are accountable for fielding questions and for being able to explain the research to anyone else, and that you are responsible for timely response. Being a coauthor can mean many things, and in order for you to get the most out of it and to feel the most useful in and so the proudest of this capacity, I include the following indications below.*

A few words on scientific writing and scientific coauthorship

Scientific authorship is not about words contributed.

*You will likely **not** contribute as many exact words or pages to the document as you have to for academic honesty in classroom assignments. Your largest expectable portion for writing yourself will likely be the Method section. However, as collaborator aiding me in collecting data and honing ideas, you are automatically and completely entitled to coauthorship. I am prepared to protect and preserve your right to authorship. However, I reserve the right to void your authorship rights in case of your actively hindering project progress or being overtly and repeatedly disrespectful. Given your junior status, it is my general policy to put you as first author(s). I understand that non-scientific sensibilities may be horrified at the idea of authorship where you have not written as much as you might normally on a classroom assignment. The sad truth is that scientific writing is an opaque skill not quickly mastered. You will have the option to revise my language, you will have the option to write your own sentences, but whether or not those sentences remain part of the draft, you remain a coauthor according to the values of scientific culture.*

Why I will send you drafts on which you are a coauthor

I expect you to bring your enthusiastic interest in what you did to the bigger problem of what other people might want to know about it. I value your intelligence, and I value as well your lack of expertise. I value you as a thoughtful person who has imperfect, incomplete knowledge. You can be an extremely valuable coauthor by pointing out to me where the text is genuinely unclear, and I expect you to be honest about what you do not understand. When papers go out for review, they go out to so-called "expert" reviewers, and they will all be unwilling and unimaginative enough to have trouble understanding what I mean. ***If you can bring your willingness and imagination to bear on what you might not understand, then your input can be extremely helpful in allowing me to learn new ways of expressing myself.*** *In other words, your strengths as a coauthor can depend as much on your knowledge as on your lack of understanding. I value your lack of understanding, and if you permit us both, we can together take my garbled vision with your lack of understanding, and if we can be honest enough with one another, we can discover new ways of saying difficult things. In that respect, I really hope you will be an active coauthor keen to drag clarity out of what I might not be saying in a way you understand yet. Coauthoring is a wonderful way for people to learn from one another, and if you can share with me your uncertainty, then we can together make science more democratic by working together to make the paper so clear that any interested undergraduate has a good shot at understanding it.*

Why I am so persistent in inviting you to be coauthor

Authorship is a way for you to show to others that you dedicated your efforts professionally and effectively to a scientific research project—apart from the relatively short-lived compensation of course credit or monetary payment. It is really, really important to me that I have this means for helping you show to your future employers/colleagues that you have this capacity to commit to a project and see it through. So, I really want to restate my excited desire that you should expect to be author and probably not last author—and that you should expect as much simply from having contributed to data collection. Also, as noted above, you and I have an opportunity to open up scientific literature to a broader audience, and I believe this opportunity aligns with the social-justice ethos of Grinnell College.

My Initials _________Your Initials____________ Date________

Coauthorship requires you to trust me

As peer review inevitably extends beyond your scheduled time working for me and probably beyond your own interest, ***I expect you trust me*** *to adapt the text to suit arbitrary/unforeseeable needs of reviewers but to remain true to the data collection procedures that you contributed, and* ***I expect you to remain confident in your author status*** *no matter my revisions. You have full right to approve every new revision.*

If you want to contribute to more words and data analysis to the paper, that is great but not a necessary criterion for inclusion as coauthor. *I will be responsible for providing feedback on your writing and how it sits—easily or uneasily—with the rather larger problem of scientific writing. You will be responsible for not taking personally what will likely be critical of your writing. I expect that you will not take this critical feedback as reflecting poorly on you as a person but rather as instruction on how odd and strange and imperfect scientific communication very often is.*

If you really, really don't want to be a coauthor, you will have to make a concerted effort to express that sentiment to me very clearly. *Why? Well, simply because, to my mind, it is extremely unethical scientific practice for me to* ***exclude*** *crucial contributors to data collection from authorship and to take credit for effort that was not mine. That this concern might weigh more heavily on different scientists' conscience is immaterial to me. It is my lab to set the conventions to protect your effort and to favor your on-paper qualifications, and it is your place to refuse sufficiently clearly and categorically.*

My Initials _________Your Initials____________ Date________

Page intentionally left blank (no need to initial)
